# Gene-Trait Matching and Prevalence of Nisin Tolerance Systems in *Lactococus lactis*

**DOI:** 10.3389/fbioe.2021.622835

**Published:** 2021-03-03

**Authors:** Lieke A. van Gijtenbeek, Thomas H. Eckhardt, Lucía Herrera-Domínguez, Elke Brockmann, Kristian Jensen, Asger Geppel, Kristian Fog Nielsen, Jannik Vindeloev, Ana Rute Neves, Gunnar Oregaard

**Affiliations:** R&D, Chr. Hansen A/S, Hørsholm, Denmark

**Keywords:** high-throughput screening, milk acidification, nisin degradation, nisin tolerance, nisin biosynthesis, NSR, *Lactococcus lactis*, gene-trait matching

## Abstract

*Lactococcus lactis* cheese starter cultures typically contain a mix of many strains and may include variants that produce and/or tolerate the antimicrobial bacteriocin nisin. Nisin is well-established as an effective agent against several undesirable Gram-positive bacteria in cheese and various other foods. In the current study, we have examined the effect of nisin on 710 individual *L. lactis* strains during milk fermentations. Changes in milk acidification profiles with and without nisin exposure, ranging from unaltered acidification to loss of acidification, could be largely explained by the type(s) and variants of nisin immunity and nisin degradation genes present, but surprisingly, also by genotypic lineage (*L. lactis* ssp. *cremoris* vs. ssp. *lactis*). Importantly, we identify that nisin degradation by NSR is frequent among *L. lactis* and therefore likely the main mechanism by which dairy-associated *L. lactis* strains tolerate nisin. Insights from this study on the strain-specific effect of nisin tolerance and degradation during milk acidification is expected to aid in the design of nisin-compatible cheese starter cultures.

## Introduction

*Lactococcus lactis* is a lactic acid bacterium widely used in the dairy industry for milk fermentation. Some *L. lactis* strains produce the Class I bacteriocin nisin, a 34 amino-acid-long peptide that shows potent antimicrobial activity against a broad range of Gram-positive bacteria (Mattick and Hirsch, 1947; Gross and Morell, 1971). Nisin is heat-resistant and acid-tolerant, largely because of a set of stable post-translational modifications (Delves-Broughton, 1996). Nisin in powder format was first introduced to the market in the 1950's as a natural product to contribute to shelf-life extensions of cheese by preventing growth of food spoilage organisms including *Clostridi*a, *Propionibacteria* and *Listeria* (Hirsch, 1951; Delves-Broughton, 1996; Molloy et al., 2011). Nisin-producing strains can be added or are naturally present in cheese starter cultures (Delves-Broughton, 1996; de Arauz et al., 2009; Ávila et al., 2014).

To match the increasing demands of the dairy industry, new starter cultures are constantly being developed by either blending traditional undefined cultures or targeted culture design (Ayad et al., 2001). Starter cultures for semi-hard cheeses like Gouda or Edam are typically composed of a mixture of lactococcal strains, including *Lactococcus lactis* ssp. *lactis* and *Lactococcus lactis* ssp. *cremoris*, and *Leuconostoc* strains (Erkus et al., 2013; Düsterhöft et al., 2017). Differences in strain composition and diversity of the starter culture impact robustness toward phage attack, acidification, eye-formation, flavor development, and rheological properties of the cheese. Hence, it is crucial to understand how the starter culture composition and subsequently the cheese quality is affected by different factors. *L. lactis* strains devoid of nisin immunity or degradation machineries are highly susceptible to nisin. We therefore hypothesized that presence of nisin may inhibit *L. lactis* cheese culture strains to different extents depending on the genetic blueprint of the strains, e.g., presence of nisin immunity and/or nisin degradation genes in each strain.

Nisin exerts multiple modes of action against bacteria. Small amounts of nisin can be sufficient to hinder cell division by binding to and subsequent displacement of lipid II molecules, which are essential precursors required for bacterial cell wall synthesis (Brötz et al., 1998; Breukink et al., 1999; Wiedemann et al., 2001; Hasper et al., 2006). When nisin concentrations increase, it assembles together with lipid II into membrane pore-forming entities (Hasper et al., 2004). At even higher concentrations, nisin molecules are also known to self-assemble into pores without the requirement for lipid II (Sahl et al., 1987; Breukink et al., 1999). Pore formation results in the dissipation of proton motive force, cytoplasm leakage including the release of autolysins, and eventually cell death (Bierbaum and Sahl, 1985; Breukink et al., 1999; MartÃnezCuesta et al., 2000). Nisin also prevents the outgrowth of bacterial spores, presumably by lipid II binding and pore formation (Egan et al., 2016 and references therein). Since nisin targets any bacterial cytoplasmic membrane including that of *L. lactis* itself, nisin-producing *L. lactis* strains co-express proteins that confer nisin autoimmunity. These are NisI, a membrane associated lipoprotein, and NisFEG, an ABC transporter (Kuipers et al., 1993; Engelke et al., 1994; Qiao et al., 1995; Siegers and Entian, 1995). Extracellular NisI binds nisin thereby abolishing pore formation (Takala et al., 2004; AlKhatib et al., 2014a; Hacker et al., 2015). Recently, it was shown that NisI also functions in cell aggregation thereby reducing accessibility to lipid II even more (AlKhatib et al., 2014a). NisFEG forms an efflux pump that translocates nisin from the membrane into the extracellular space (Stein et al., 2003). NisI and NisFEG have a synergistic effect and enable *L. lactis* cells to tolerate high levels of nisin (up to 700 nM, ~2.35 mg L^−1^), which drops to 10–30% when expressed separate from each other (Kuipers et al., 1993; Qiao et al., 1995; Duan et al., 1996; Ra et al., 1996, 1999; Stein et al., 2003; AlKhatib et al., 2014a,b).

Nisin production and immunity by *L. lactis* strains are established by a conserved biosynthesis gene cassette consisting of four transcriptional units, *nisABTCIPRK, nisI, nisRK*, and *nisFEG* (Kuipers et al., 1993; Ra and Saris, 1995; de Ruyter et al., 1996; Ra et al., 1996; Li and O'Sullivan, 2006; Trmčić et al., 2011) The *nisA* gene encodes the nisin bacteriocin of which several variants have been reported (A, Z, F, and Q) in *L. lactis* (Mulders et al., 1991; De Kwaadsteniet et al., 2008; Fukao et al., 2008). The transcription of the nisin biosynthesis and autoimmunity operons (*nisABTCIPRK* and *nisFEG*) is activated through a nisin-mediated positive feedback loop conducted by the two-component system NisRK (Kuipers et al., 1995; de Ruyter et al., 1996; Kleerebezem et al., 1997). Due to this positive autoregulation, strains that carry the complete nisin biosynthesis cassette are locked in a nisin-producing state. The two other transcriptional units, *nisI* and *nisRK*, are driven by a weak and a relatively strong constitutive promoter, respectively, that function in the absence of nisin (de Ruyter et al., 1996; Li and O'Sullivan, 2006). However, both are believed to be trivial in nisin-producing strains as transcription from the *nisA* promoter overrules the effect of the internal operator sites.

In addition to *nisI* and *nisFEG*, a third nisin resistance determinant (*nsr*) was mapped to certain plasmids in non-nisin producing lactococci (McKay and Baldwin, 1984; Froseth and McKay, 1991; Liu et al., 1997). Already in 1984, it was suggested that the product of this gene might function as a nisinase, a nisin degrading enzyme previously discovered in *Streptococcus thermophilus* (Alifax and Chevalier, 1962). Over a quarter of a century later, it was confirmed that NSR is a tail-specific membrane-bound protease that cleaves nisin Z after the 28th position (Sun et al., 2009). This renders the remaining peptide close to inactive through a significant reduction in membrane affinity and pore-forming capacity (Sun et al., 2009). Over the years, similar membrane-associated proteases have been identified in Gram-positive species (Khosa et al., 2013; Draper et al., 2015). One of these is the well-characterized *Streptococcus agalactiae* nisin resistance protein SaNSR of which its structure has been resolved (Khosa et al., 2015). While the IC_50_ of a nisin-sensitive *L. lactis* strain overexpressing NisI is 5-to-10-fold greater than the wild type, SaNSR overexpression in the same cells enabled cells to withstand up to 18-to-20-fold more nisin (Khosa et al., 2013).

To investigate the potential effect of nisin on *L. lactis* strains, we initiated a study directed at evaluating the effect of nisin on the individual acidification profiles of 710 individual *L. lactis* strains, from which many are derived or used in common starter cultures. By genome sequencing and PCR, we correlated the observed acidification profiles with the presence or absence of nisin immunity and degradation genes, concurrently giving insights in the collection-wide distribution of such elements. Furthermore, nisin production and degradation was measured using a newly developed high-throughput HPLC-MS/MS method, which enabled us to elucidate the nisin degradation capacity of each strain during milk fermentations.

## Materials and Methods

### Strains and Growth Conditions

A total of 710 proprietary *L. lactis* strains originating from the Chr. Hansen Culture Collection were used in this study, for details see Supplementary Table 1. The strains were routinely grown as standing cultures in Oxoid M17 (Thermo Fisher Scientific, Waltham, MA, USA) with glucose 0.5% (w/v) (GM17) or a mixture of 1% (w/v) glucose and 1% (w/v) lactose (GLM17) at 30°C for 18 h.

### Genome Sequencing and Analyses

All 710 strains were subjected to whole-genome sequencing on an Illumina MiSeq, yielding reads of 250 bases. The reads were assembled into contigs using CLC Genomics Workbench (Qiagen, Århus, Denmark). Contigs with a mean depth of coverage <0.25 of the total mean depth of coverage were discarded as contaminants. A gene search was conducted in all 710 genomes to map the presence of genes encoding nisin-related proteins. The query sequences used in the gene search can be found in Supplementary Table 2. The genomes were searched using blastn and tblastn for nucleotide sequence and protein sequence queries, respectively, with an *E*-value cut-off of 0.01. A gene was considered present in a genome if a hit with more than 90% query coverage and 80% identity was found. After observing nisin degradation in strains where no *nsr* gene was detected, it was found that some strains had shorter versions of *nsr*. To identify such variants, we repeated the tblastn search for the *nsr* protein sequence while reducing the query coverage threshold to 20% (Schliep, 2011). Whole genome k-mer trees [K-mer length: 16, prefix: AT, distance function: Feature Frequency Profile *via* Jensen-Shannon divergences (FFP)] of the 710 draft genomes and 219 RefSeq genomes were calculated using the Microbial Genomics module of the CLC Genomics workbench. A newly developed MLST scheme was employed including the household genes *dnaK, fusA, groEL, gyrA, gyrB, ileS, lep, pheS, recA, rpoA, rpoB*, and *rpoC* (see Supplementary File 1 for sequences of indicated genes for all strains). Concatenated sequences of the twelve genes were used to calculate a maximum likelihood tree with the Phangorn package I (Schliep, 2011) in R software. Due to quality requirements, a total of 206 instead of 219 *L. lactis* RefSeq genomes were taken along in the MLST analysis. Both k-mer and MLST trees were plotted with iTOLv5.5 (Letunic and Bork, 2019).

### Milk Acidification Profiles With and Without Nisin

Cell-free supernatants (CFS) with or without nisin were obtained from 500 mL GLM17 cultures of *L. lactis* well-studied strains ATCC11454 or Wg2, respectively, by centrifugation for 5 min at 5,000×*g*. Collected supernatant was adjusted to pH 6.0 by the addition of 0.25 M NaOH, filter-sterilized using a Minisart® 0.22 μm filter (Sartorius, Göttingen, Germany) and stored at −80°C in 10 mL aliquots. CFS of Wg2 yielded 0.0 μg ml^−1^ nisin A, whereas CFS of ATCC 11454 yielded 6.1 ± 0.8 μg ml^−1^ nisin A, as measured using the HPLC-MS/MS method detailed below. The two CFS types were subsequently used to evaluate the milk acidification profiles of the 710 *Lactococcus* strains as follows. A volume of 20 μl fresh overnight culture of each *L. lactis* strain grown in GLM17 in a 96-wells microtiter plate was used to inoculate 1,980 μl prewarmed (30°C) pasteurized semi-skimmed milk supplemented with 0.2% (w/v) yeast extract and 5% (v/v) pH indicator solution (1 g L^−1^ Bromocresol Purple sodium salt (Sigma Aldrich, St. Louis, MS, United States); 1 g L^−1^ Bromocresol Green sodium salt (Sigma Aldrich), pH 7.0, filtered-sterilized). One hundred and fifty microliter of each of the 710 inoculated milk samples were then mixed with 50 μl of one of the two CFS types described above, resulting in a final nisin A concentration of 0.0 or 1.5 μg ml^−1^, and incubated for 18 h at 30°C on flatbed scanners (HP ScanJet G4010) to obtain HUE-values every 6 min. Acidification profiles were obtained by converting HUE-values into pH values as described previously (Poulsen et al., 2019). Experiments with CFS from Wg2 and ATCC 11454 were always run in parallel and the experiments were performed in triplicates: one technical and two biological replicates.

### Analysis of Milk Acidification Profiles

As mentioned above, inoculation material for the milk acidifications originated from overnight cultures with different biomass and pH, which caused small changes in both initial and end pH, as well as the time for the acidification to start. Therefore, in order to define robust thresholds for phenotypic classification, the acidification curves were first normalized to the same maximum (initial) pH of 6.4. The pH values were re-scaled by setting the minimum and maximum pH for each pair of curves per strain to 0 and 1, respectively. Next, curves were also normalized in time so that starting timepoint of each pair of acidification curves was the same across replicates. The starting time was calculated per replicate using the milk acidification curve that had no nisin. This starting point was then subtracted across all timepoints from each pair of curves. Finally, absolute change in pH, area under the curve and starting timepoint for each curve and condition were calculated and averaged across replicates. These parameters were used to define the four different phenotypes. For detailed description on the data processing steps see Supplementary Table 3. Data and statistical analyses were performed using the computing environment R. Raincloud plots were made according to Allen et al. (2019). The central matrix layout was used using the UpSet package (Gu et al., 2016).

### Detection of Nisin A and Nisin^1−28^ by HPLC-MS/MS in Culture CFS

To set up a detection method for full-length and NSR-degraded nisin A, CFS was collected from *L. lactis* strains CH-1 (*nisA–, nsr*+) and ATCC 11454 (*nisA*+, *nsr–*) grown in GM17 spiked with 0.9 μg ml^−1^ nisin A (Chrisin®, Chr. Hansen A/S, Denmark). Chrisin® was shown to be 2.26% pure when compared to nisin A from Sigma-Aldrich (defined as 2.5% by FAO, 2013). Sterile GM17 spiked with 0.9 μg/ml nisin A was taken along as a blank control sample. A 30 μL sample from each CFS was then mixed in 1.5 mL Eppendorf tubes with 870 μL extraction buffer to which nisin Q was introduced as an internal standard (IS) to improve the precision of the assay. Nisin Q was chosen to get: (i) an as similar molecule to nisin A as possible; (ii) an IS not interfering with nisin A measurements; and (iii) an IS also susceptible to NSR degradation. The extraction buffer containing nisin Q was made as follows: CFS collected from a culture of the nisin Q-producing CH-5 strain grown in GM17 was quenched by adding 3% (v/v) acetonitrile (ACN) and 1% (v/v) formic acid (FA). 1.5 ml of this extract was then added to 100 ml of extraction buffer consisting of 100 mg/L Bovine Serum Albumin (BSA; A-2153, Sigma), 20% (v/v) ACN and 0.5% (v/v) FA dissolved in MilliQ water. The extracts were transferred to polypropylene HPLC vials, and nisin A, nisin Q, and nisin^1−28^ (nisin A degraded by NSR) levels were analyzed on a binary ACQUITY UPLC system (Waters, Milford, MA, USA) equipped with a sample organizer (held at 8°C) and connected to a Xevo TQ-XS Triple Quadrupole Mass Spectrometry (MS) instrument (Waters) equipped with an Electrospray source operated in positive mode. One microliter subsamples were injected onto a PLRP-S 300 Å, 2.1 × 150 mm, 3 μm HPLC column (Agilent Technologies, Waldbronn, Germany) held at 60°C and eluted using a linear gradient of 25–40% (v/v) ACN with 0.1% (v/v) FA using a flow rate of 0.5 mL min^−1^. The following MS conditions were applied: Capillary voltage: 3 kV, collision gas (Ar), desolvation temperature: 550°C, desolvation gas flow: 1,100 L/h, cone flow: 150 L/h, nebulizer: 6 bar. For all analytes the cone was held at 40 V and a fragmentation energy of 16 eV. Waters TargetLynx Software was used for data analysis and peak integration. The following multiple reaction monitoring (MRM) fragmentations, with a dwell time of 20 ms, were used for detection: (1) the [M+5H]^5+^ ion of nisin A from *m/z* 671.7 to 811.2, (2) the [M+5H]^5+^ ion of nisin Q from *m/z* 666.2 to 804.4, and (3) the [M+4H]^4+^ ion of nisin^1−28^ *m/z* 680.55–869.7). Nisin^1−28^ eluted ~0.35 min later than nisin A but 0.29 min earlier than nisin Q. From calibrants with constant nisin Q and variable nisin A concentrations, the nisin A peak areas, divided by the peak area of the nisin Q peak area, was used to determine the nisin A concentrations. From a degradation of nisin A with cell free NSR culture extract, the response factor difference between nisin A and nisin^1−28^ was estimated to approximate nisin^1−28^ concentrations in ATCC 11454 and CH-1 CFS. This method was also used to obtain the nisin A concentrations in the CFS of ATCC 11454 and Wg2 added during the milk acidification experiment.

### High-Throughput Detection of Nisin Degradation and Nisin A Production by *L. lactis* Strains

Nisin degradation and nisin A production by each of the 710 *L. lactis* strains was assessed by measuring the decrease or increase of nisin in samples spiked with known concentrations of nisin, using a high-throughput HPLC-MS/MS as follows. Two milliliter of skimmed milk supplemented with 0.9 μg ml^−1^ nisin A and 0.2% (w/v) yeast extract was inoculated with 20 μl of a *L. lactis* GLM17 overnight culture and incubated in 2.5 mL deep well plates for 18 h at 30°C and stored at −20°C until further analysis. As a control, non-inoculated skimmed milk samples, to which 0.9 μg ml^−1^ nisin A was added, were taken along. Thirty microliter from each of the thawed samples was extracted with 870 μL extraction buffer in a 1 mL microtiter deep well plate. The plates were shaken for 1 h on an orbital mixing table, left overnight at 5°C and centrifuged at 6,000×*g* for 30 min, after which the samples were loaded for HPLC-MS/MS as described in the method above, except that a MRM for nisin Z was also included at *m/z* 667.2–739. Due to variations in the control samples from plate to plate, nisin concentrations in control samples were set to 1 AU mL^−1^ and nisin concentrations in fermented milk samples were converted to ratios of this arbitrary control value. Since we added a known concentration of nisin A, a decrease or increase of nisin A levels was used as a proxy for degradation and production, respectively. Degradation of nisin was confirmed in a subset of samples by measuring the accumulation of the NSR degradation product with the method described in the section above (Supplementary Table 1).

### Re-evaluation of Strains With a Genotype/Phenotype Discrepancy

Strains with a *nsr–* genotype that displayed a nisin degrading phenotype, as well as strains where the *nsr* gene was found on the edge of a contig, were individually examined for the presence of the *nsr* gene using a colony PCR approach with REDTaq DNA polymerase Master Mix (VWR, Radnor, PA, USA) and primers annealing to two conserved regions within the *nsr gene* of *L. lactis* strains (oLIGI021 and oLIGI022), ranging from nucleotide 112–901 of the 958-nts long *nsr* gene. A total of 46 strains were tested by colony PCR using oLIGI021 and oLIGI022 to confirm the presence of a copy of the *nsr* gene. Of the 15 strains in which the *nsr* gene was detected at the edge of a contig in the genome sequence, 14 strains resulted in a PCR product validating the presence of *nsr*, while one strain contained an estimated insertion of roughly 6 kB in its *nsr* gene. The 31 remaining strains, corresponding to those in which the *nsr* gene could not be detected in the genome sequence but that showed signs of nisin degradation, 25 were confirmed to carry the *nsr* gene. Primer sets to confirm several genotypes were oLIGI023/24 for *nisA*, oLIGI043/44 for *nisI*, oLIGI045/46 for *nisF*. An overview of primers used in this study can be found in Supplementary Table 4.

### *nisA* and *nsr* Mutant Generation

For targeted gene deletions in strain CH-2, the pCS1966/*oroP* system was employed (Solem et al., 2008). Standard molecular cloning techniques were performed essentially as described (Sambrook et al., 1989). Flanking regions of either *nisA* or *nsr* gene were amplified using Q5 DNA polymerase (NEB, Ipswich, MA, USA) from CH-2 chromosomal DNA with primer pairs oLIGI003/oLIGI004 and oLIGI005/oLIGI006 or primer pairs oLIGI014/oLIGI015 and oLIGI016/oLIGI017, respectively. The backbone of pCS1966 was amplified using oLIGI001 and oLIGI002. Fragments were assembled using NEBuilder® HiFi DNA Assembly Master Mix (NEB) and transformed into competent *E. coli* DH5α cells (NEB), yielding pLIGI001 and pLIGI003. Purification of DNA fragments was done using the Monarch® PCR & DNA Cleanup and DNA Gel Extraction Kits (NEB) and plasmids were isolated using the NucleoSpin® Plasmid kit (Macherey-Nagel, Düren, Germany). pLIGI001 was then used to delete *nisA*, whereas pLIGI003 was used to delete *nsr*, both as described previously (Solem et al., 2008). Electrocompetent *L. lactis* cells were transformed using electroporation with a Bio-Rad Gene Pulser (Bio-Rad Laboratories, Richmond, CA). PCR amplification on colonies with REDTaq DNA Polymerase Master Mix (VWR) was used for routine checks for correct DNA constructs.

### Well Diffusion Assays

For well diffusion assays, strains ATCC 11454, CH-1, CH-2, CH-3, CH-4, mutant strains CH-2 Δ*nisA* and CH-2 Δ*nsr*, and the nisin-sensitive MG1363 strain were grown to stationary phase in 16 h from single colonies in GM17. CFS of all strains except MG1363 was collected by centrifugation for 10 min at 4,000×g and subsequent filter-sterilization of the supernatant. The MG1363 culture was diluted 1,000 times in fresh GM17 broth, mixed 1:1 with GM17 agar and poured as a soft agar layer while leaving out 10 mm holes that were filled with 200 μL of the collected CFS samples. The assay was incubated at room temperature until halos were visible. For measurements of nisin A and the nisin^1−28^ fragment in GM17, the cultures were grown as described above and stored for 24 h at −80°C before CFS was collected using 0.22 μm 96w filter plates (Acroprep, Pall laboratory, Ann Arbor, MI). These samples were then subjected to HPLC-MS/MS analysis using the extraction and analysis procedure as described above.

## Results

### Effect of Nisin on Milk Acidification by Individual *L. lactis* Strains

**Figure 1:**
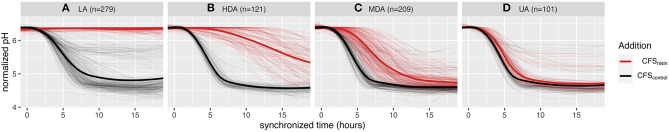
Effect of nisin on milk acidification profiles and phenotypic classification of 710 *L. lactis* strains. Milk acidification curves of strains grown at 30°C without nisin (CFS_control_) and with 1.5 μg ml^−1^ nisin addition (CFS_nisin_) The thin lines depict the averaged milk acidification data of three replicates while the thicker line depicts the averaged curves per group. The nisin-containing milk phenotypes are classified to four groups; **(A)** LA, loss of acidification; **(B)** HDA, highly delayed acidification; **(C)** MDA, mildly delayed acidification; and **(D)** UA, unaltered acidification. All acidification curves are synchronized based on acidification onset of each respective CFS_control_ profile and normalized to a start pH of 6.4.

A total of 710 *L. lactis* strains were monitored, in a time-resolved fashion, to evaluate their ability to acidify milk in the presence of nisin (CFS collected from the nisin producing *L. lactis* strain ATCC 11454, CFS_nisin_) or in absence of nisin (CFS collected from the non-nisin producing *L. lactis* strain Wg2, CFS_control_). Without nisin, all strains were able to acidify milk in <18 h: the majority (88%) of the strains were able to acidify milk from a start pH of 6.4 down to a pH value below 5.0, while the remaining strains acidified milk to an end pH between 5.0 and 6.0 instead (Supplementary Table 1). The addition of CFS_nisin_ (final concentration of nisin A in milk of 1.5 μg ml^−1^), led to a wide variation of acidification profiles, ranging from unaltered acidification to loss of acidification. In order to get an impartial comparison of the obtained milk acidification profiles in the presence or absence of nisin for each separate strain, the start and end pH were normalized to correspond to values 1 and 0, respectively, and the curves were synchronized so that the control curves would start acidifying at the same timepoint. From each normalized acidification curve the absolute change in pH (ΔpH) was derived, and the difference in area under the curve (ΔAUC) and acidification starting time points (Δtime_0_) between CFS_nisin_ and CFS_control_ treated conditions for every strain were calculated. After multiple attempts for classification, we noticed that ΔAUC alone could not allow for a clear separation of the phenotypes. Instead, combining these three parameters (ΔpH, ΔAUC, Δtime_0_) allowed us to classify strains into four distinct phenotypic groups (rules are described in Supplementary Table 3). We define the resulting phenotypic groups as (A) loss of acidification (**LA**), (B) highly delayed acidification (**HDA**), (C) mildly delayed acidification (**MDA**), and (D) unaltered acidification (**UA**) (Figure 1 and Supplementary Figure 1).

When milk fermentations were carried out in the presence of CFS_nisin_, 279 *L. lactis* strains clustered to the LA phenotype, indicating that these strains are sensitive to nisin (Figure 1A). A total of 101 *L. lactis* strains grouped to the UA phenotype, for which both the acidification rate and depth remained mostly unchanged upon the addition of CFS_nisin_ compared to addition of CFS_control_ (Figure 1D). For the remaining strains, the effect of nisin resulted in a range of delays in the onset of acidification. Of these, 121 strains clustered to the HDA and 209 to the MDA phenotypes (Figures 1B,C). We identified a small set of strains that did not show a delay but a change in acidification depth as a result of nisin addition, which therefore also clustered to the mild (MDA) phenotypic group, see also Supplementary Figure 2A.

### Diversity of the *L. lactis* Strain Collection

**Figure 2:**
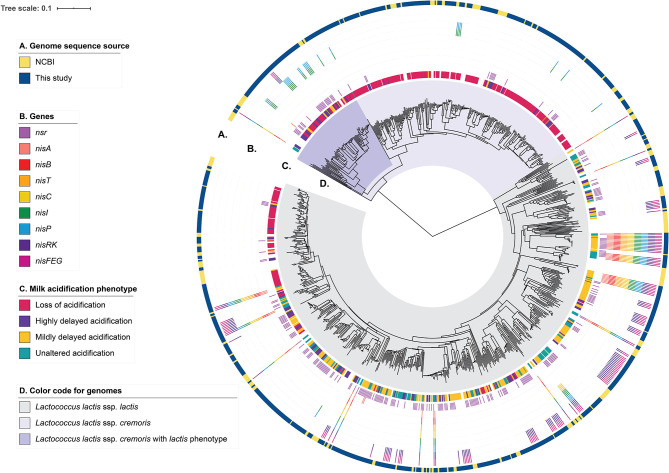
Genotypic diversity of the *L. lactis* strains. A *k*-mer tree depicting the genotypic relatedness of 929 *L. lactis* ssp. *cremoris* and ssp. *lactis* strains. Indicated from outer to inner ring are: **(A)** Strain-specific genome sequence origin, obtained either from the 710 draft genomes from this study or the publicly available 219 *L. lactis* RefSeq genomes. **(B)** Strain-specific presence of nisin-related genes or gene clusters. **(C)** Strain-specific milk acidification phenotypes. **(D)** Subspecies clusters of *L. lactis* ssp. *lactis* and *L. lactis* ssp. *cremoris*. The latter is further divided into whether ssp. *cremoris* strain is predicted to have a *cremoris* or a *lactis* phenotype.

In order to assess the genotypic diversity, the strains in the collection were whole-genome sequenced and their genomic relatedness assessed by a k-mer-based and a MLST-based tree construction (Figure 2 and Supplementary Figure 3). From this analysis, 64% of the strains (*n* = 457) belong to *L. lactis* ssp. *lactis* and the remaining 36% (*n* = 253) of the strains belong to *L. lactis* ssp. *cremoris*. We compared our genomes to 206 unique lactococcal assemblies publicly available (July 2020) in NCBI (National Center for Biotechnology Information) RefSeq database of which 73% and 26% map to *L. lactis* ssp. *lactis* and ssp*cremoris*, respectively (Figure 2 and Supplementary Figure 3). Furthermore, the comparison shows that our strain collection spans the full spectrum of genomic diversity represented by the publicly available *L. lactis* genomes with some smaller lineages exclusively represented in our collection and to a lesser extent vice versa. We subsequently searched the genome sequences of our collection for orthologous groups of protein sequences (OGs) to capture ssp. *cremoris* with a reversed phenotype e.g., strains that display a ssp. *lactis* phenotype while having a ssp. *cremoris* genotype, as previously reported (Kelleher et al., 2017; Wels et al., 2019). This analysis showed that 25% of the ssp. *cremoris* strains in our collection carry sequences corresponding to OGs specific for ssp. *cremoris* strains with a ssp. *lactis* phenotype (Figure 2). Accordingly, these strains cluster in one of the two ssp. *cremoris* lineages, together with previously identified ssp. *cremoris* strains with a ssp. *lactis* phenotype such as KW2, N41, V4, MG1363, and NCDO763 (Wels et al., 2019).

### Effect of *nsr, nisFEG*, and *nisI* on Milk Acidification in the Presence of Nisin

To better understand how the presence of genes for nisin biosynthesis, nisin immunity and/or nisin degradation correlate to the observed variability in milk acidification upon addition of nisin, we performed gene-trait matching of nisin-related genes to observed acidification phenotypes. The distribution of genes encoding key proteins involved in nisin biosynthesis (a nisin structural gene: *nisA*; the nisin biosynthetic machinery: *nisB, nisT, nisC*, and *nisP; nis* gene regulation: *nisR* and *nisK*), nisin immunity (*nisI* and *nisFEG*) and nisin degradation (*nsr*) within the 710 strains along with the milk acidification phenotypes (LA, HDA, MDA, UA) are visualized in Figure 2 and Supplementary Figure 1. It is apparent that most ssp. *cremoris* have the LA phenotype, whereas the ssp. *lactis* have more variable phenotypes, with many strains belonging to UA, MDA, and HDA.

#### Complete Nisin Gene Cassette vs. Acidification

**Figure 3:**
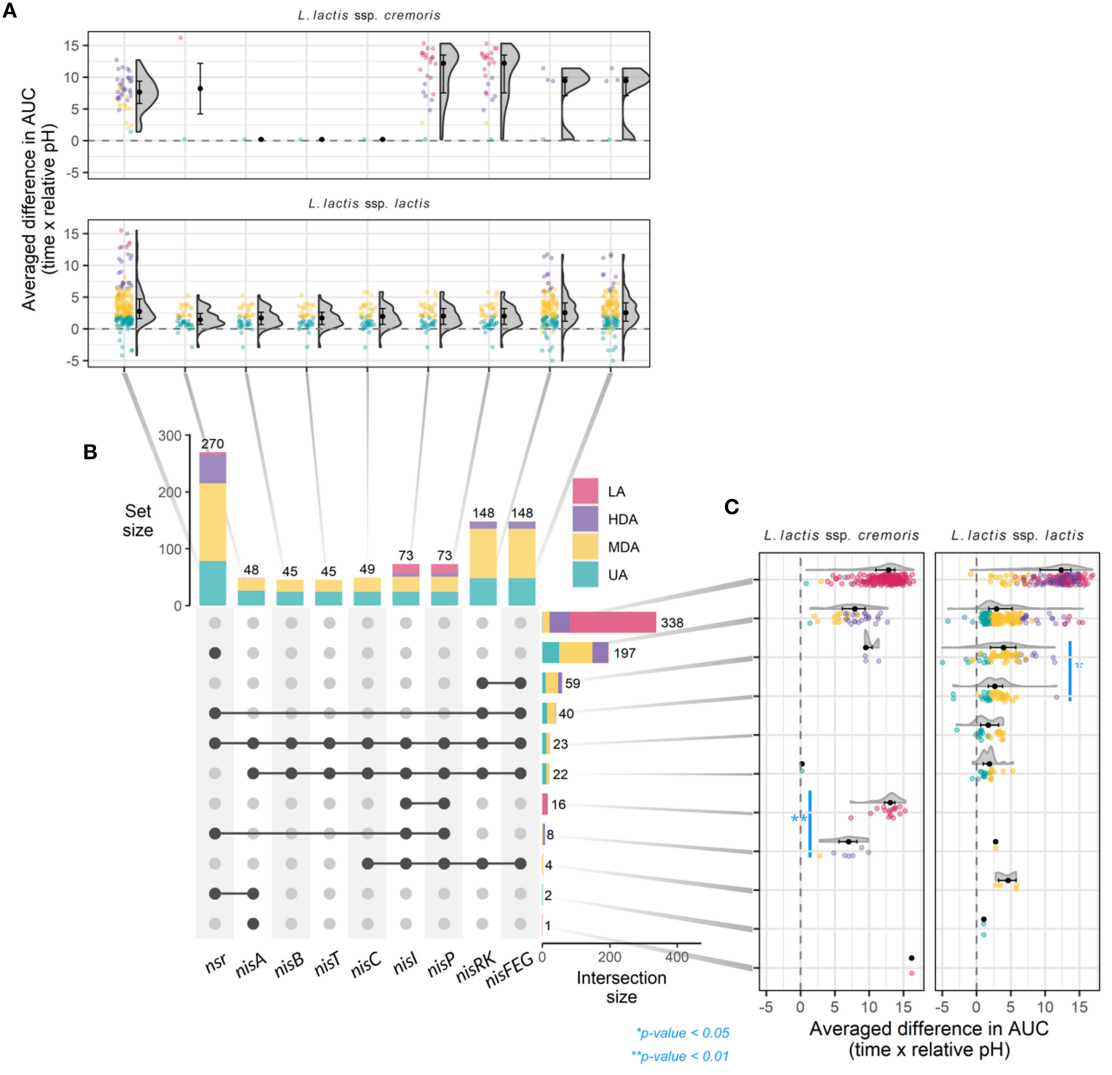
Gene-trait matching of nisin tolerance genotypes with milk acidification patterns. **(A)** Raincloud plots depicting the averaged ΔAUC of every strain for milk acidification curves with and without nisin, grouped by individual nisin-related gene and subspecies. **(B)** Matrix layout showing individual presence (vertical) and overlap in (horizontal) nisin-related genes in 710 *L. lactis* strain and the respective distributions of phenotypes and total number of strains that contain the indicated gene or set of genes. Dark dots connected with solid lines indicate genes present in a group, while gray dots indicate gene absence. **(C)** Raincloud plots depicting ΔAUC values as in **(A)**, but now grouped by genotype for nisin-related genes and subspecies. For all raincloud plots, black dots represent the median values whereas black lines mark the boundaries for the center 50% of the distribution. *P*-values were calculated using the Mann-Whitney-Wilcoxon test.

A total of 45 strains (6.3%) contain the full nisin gene cluster required for nisin biosynthesis (*nisABTCIPRK-FEG*). In fact, 24 of these strains acidified milk without any delay in the presence of CFS_nisin_, and were classified to the UA phenotypic group, while 21 do so with only a mild delay or higher end pH and therefore classified to the MDA phenotypic group (Figure 3 and Supplementary Figure 2). Out of 45 strains, 44 belong to the ssp. *lactis* group. The single ssp. *cremoris* strain that contains the nisin gene cassette groups to the genotype *cremoris*/phenotype *lactis* clade and is one out of only three ssp. *cremoris* strains that are not affected by the addition of nisin (Wels et al., 2019) (see also Figure 2).

Interestingly, of all strains that contain a full nisin biosynthesis gene cluster, 23 also contain *nsr* (Figure 3 and Supplementary Figure 1). We therefore wondered if the presence of both *nsr* and immunity genes would allow for faster acidification than that of only the immunity genes. We found a bimodal distribution in the acidification delays for the former group of strains indicating that two subgroups exist, one containing strains that acidify better than the strains with just immunity and a second, less tolerant group (Figure 3). Interestingly, the two subgroups appeared to have different variations of the *nsr* sequence (variant 25 for the UA group and variant 17 for the MDA group), putatively yielding proteases with different activities (Supplementary Table 1 and Supplementary Figure 6D).

To summarize, the current data confirms previous reports that the presence of nisin immunity genes *nisI* and *nisFEG* together constitute a strong mechanism in establishing nisin tolerance. Moreover, we find that the full nisin gene cluster is a feature mainly present in ssp. *lactis*.

#### Partial Nisin Gene Cassette vs. Acidification

Many strains exist that do not possess the full subset of genes for nisin biosynthesis and immunity but, instead, only contain one nisin immunity component (*nisI* and/or *nisFEG*) (Figure 3 and Supplementary Figure 1) (Wels et al., 2019). Four ssp. *lactis* were identified that contain genes for full nisin immunity (*nisIP-RK-FEG*) but have lost the corresponding synthesis genes. All show an MDA phenotype. A total of 59 strains contain *nisRK-FEG* without the rest of the nisin gene cassette. Like reported previously, we find that *nisFEG* genes always co-occur with *nisRK* (Wels et al., 2019). The three ssp. *cremoris* strains that possess unaccompanied *nisRK-FEG* display a HDA phenotype, while the 56 ssp. *lactis* strains appear divided over HDA (9), MDA (37), and UA (10) phenotypes (Figure 3 and Supplementary Figure 1). This again indicates that ssp. *lactis* strains with only *nisRK-FEG* are coping better with nisin than ssp. *cremoris* strains with the same set of immunity genes. Furthermore, *nisRK*-*FEG* genes are more prevalent in ssp. *lactis* than in ssp. *cremoris*. As reported previously, the *nisI* gene, when found without the remainder of the nisin biosynthesis cassette, typically still co-occurs with *nisP* (Tarazanova et al., 2016; Wels et al., 2019). In our collection, *nisIP* is uniquely present in 16 ssp. *cremoris*, all displaying LA (Figure 3). In contrast to these findings, NisI was previously reported to deliver substantial, but not full immunity against nisin in the absence of NisFEG (AlKhatib et al., 2014a; Tarazanova et al., 2016). NisI is believed to interact in an equimolar stoichiometry with nisin molecules (Hacker et al., 2015). This could lead to a significant surplus of nisin molecules under the tested conditions here in which a high concentration of nisin was employed. In order to test if NisI alone has the capacity to protect the cells against nisin, albeit at lower levels, the acidification experiments were repeated for these strains in the presence of 0.2 μg ml^−1^ nisin (Supplementary Figure 4). We did not detect an improvement in all but one of the acidification profiles in the presence of lower levels of nisin.

In short, strains with NisFEG alone are mostly ssp. *lactis* and can cope relatively well in the presence of nisin, whereas strains that only possess NisI are uniquely ssp. *cremoris* and do not. Interestingly, the average difference in AUC is slightly less in *nsr*+ strains compared to *nisFEG*+ strains, and even more so in *nsr*+*/nisFEG*+ strains, indicating that the presence of the *nsr* gene confers better tolerance toward nisin, and that NSR thus is a stronger protective force than the individual immunity systems from the nisin gene cassette (Figure 3).

#### *nsr*+ Genotype vs. Acidification

A striking 270 *L. lactis* strains (38%) possess a gene encoding the nisin protease NSR (Figure 3) of which the majority (98%) were able to acidify milk, albeit with a wide window of delays (UA: 78, MDA: 137, and HDA: 51 and LA: 4). Of these, 197 of the strains (73%) contain only *nsr* and none of the immunity genes (Figure 3). The fact that 149 strains with *nsr*+ genotype and no nisin immunity genes display a UA or MDA phenotype clearly demonstrates the importance of NSR for nisin tolerance. The milk acidification profiles of the strains that only have the *nsr*+ genotype differ greatly between ssp. *lactis* and ssp. *cremoris* strains, with the former showing generally better acidification in the presence of nisin (Figure 3C and Supplementary Figure 5). In summary, it is evident that there is large phenotypic variation between *nsr*+ strains in terms of nisin tolerance. The general trend is that strains with only *nsr* are able to acidify milk in the presence of nisin, albeit with different delays in the onset of acidification. It could be that the underlying differences in genetic make-up and/or post-transcriptional regulation between ssp. *cremoris* and ssp. *lactis* play an important role in further determining nisin tolerance.

#### Strains Without Nisin Immunity or *nsr* Genes vs. Acidification

A total of 338 strains did neither contain *nsr, nisI* nor *nisFEG*. As expected, the majority of these strains (258; 76%) did not acidify milk in the presence of nisin. However, 1, 21, and 58 strains mapped to the UA, MDA, and HDA phenotypic group, respectively (Figure 3 and Supplementary Table 1). As these phenotypes deviate from the predicted LA phenotype of a strain with a *nsr- nisI- nisFEG-* genotype, we confirmed the absence of nisin immunity and degradation genes using a colony PCR. Therefore, the reason behind the UA, MDA, and HDA phenotypes of these strains remains elusive.

### Effect of *nsr+* Strains on Nisin Degradation

**Figure 4:**
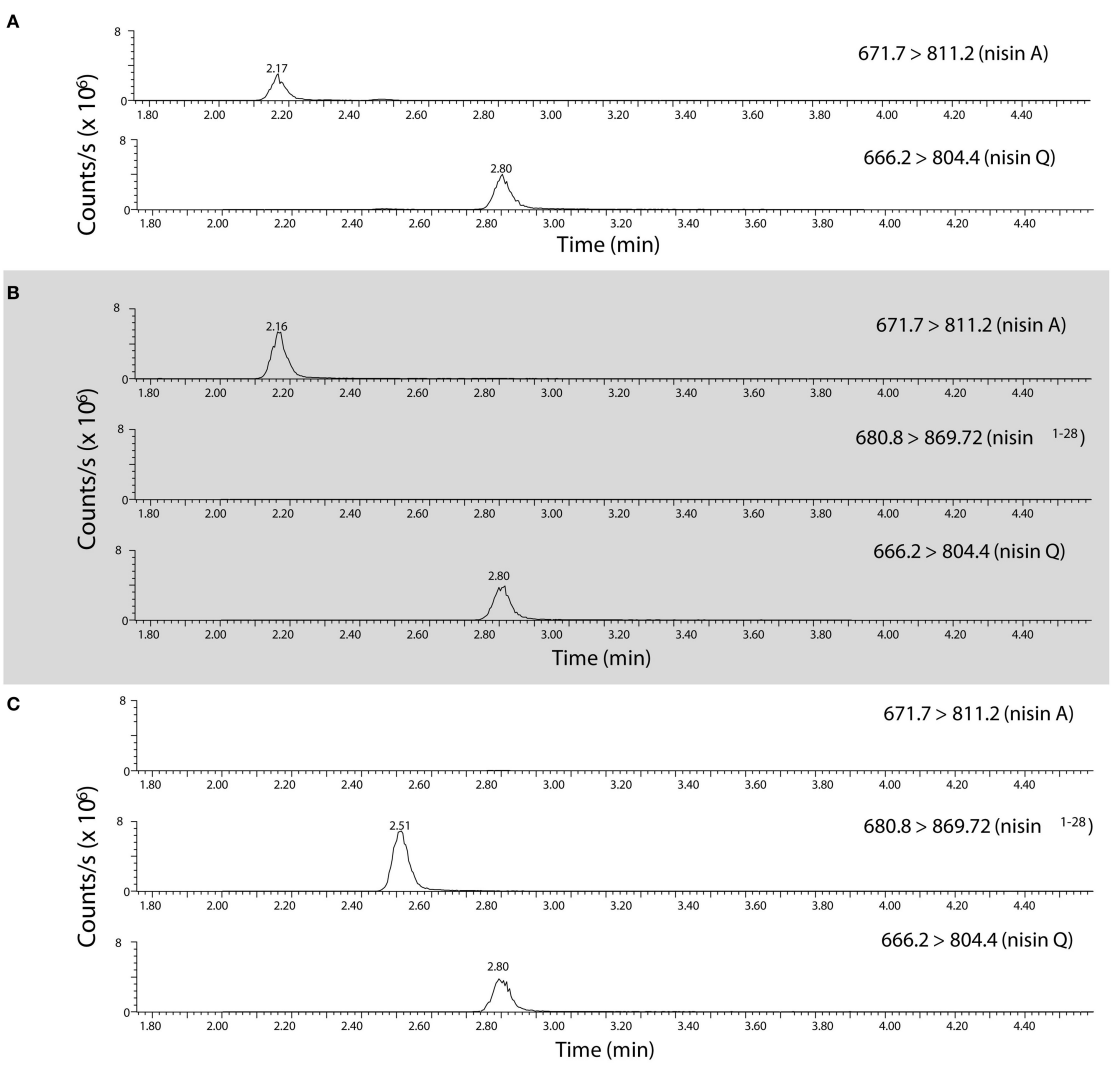
Identification of nisin A, nisin Q, and nisin^1−28^ in CFS using high resolution mass spectrometry. HPLC-MS/MS MRM transitions of quantification ions for nisin A [the [M+5H]^5+^ ion from *m/z* 671.7 to 811.2], nisin Q [the [M+5H]^5+^ ion of nisin Q from *m/z* 666.2 to 804.4,] and nisin^1−28^ [the [M+4H]^4+^ ion from *m/z* 680.55 to 869.7] as detected in **(A)** a control mix of nisin A and nisin Q, **(B)** CFS collected from CHCC 11454 (*nisA*+*, nsr-*) and **(C)**, CFS collected from CH-1 (*nsr–, nsr*+). Note that the parent isolation window of the [M+4H]^4+^ ion of nisin^1−28^ (*m/z* 680.55) was moved *m/z* 0.25 as the A+1 isotopomer was the most abundant, creating the MRM transition 680.8 → 869.7.

Since a high fraction of *nsr*+ strains were tolerant to nisin in milk, we were interested to find out to what extent nisin can be degraded by distinct *nsr*+ strains. To study the impact of each strain on nisin degradation in more detail, we first validated the role of lactococcal NSR in nisin A degradation. To accurately measure nisin A and detect its NSR degradation product, an assay based on high resolution mass spectrometry was developed. It was previously shown that nisin Z is cleaved after position 28 by the NSR enzyme thereby creating a large and a small fragment, which for nisin A should result in two fragments with monoisotopic masses of 2718.1854 Da (nisin^1−28^) and 869.3752 Da (nisin^29−34^), respectively (Sun et al., 2009). We initially identified nisin^1−28^ in CFS of CH-1(*nis–, nsr*+) incubated with nisin A by high resolution mass spectrometry on a HPLC-QTOF instrument (data not shown). Using a HPLC-MS/MS Triple Quadrupole Mass spectrometer, MS/MS fragmentation of CFS eluates collected from ATCC 11454 (*nisA*+, *nsr–*) and CH-1 (*nsr–, nsr*+) strains grown in the presence of nisin A and later on spiked with nisin Q as an internal standard led to the identification of quantification ions specific for nisin A, nisin Q, and nisin^1−28^ (Figure 4). In CFS of ATCC 11454, an increase in nisin A can be detected over the nisin A-spiked control sample. In contrast, no nisin A could be detected in the CFS derived from the *nsr*+ strain CH-1. However, in the latter, a fragment corresponding to the large NSR degradation product nisin^1−28^ could be accurately detected. This confirms that the lactococcal NSR is able to degrade nisin A. This was then used to measure nisin A and detect its NSR degradation in all further samples.

**Figure 5:**
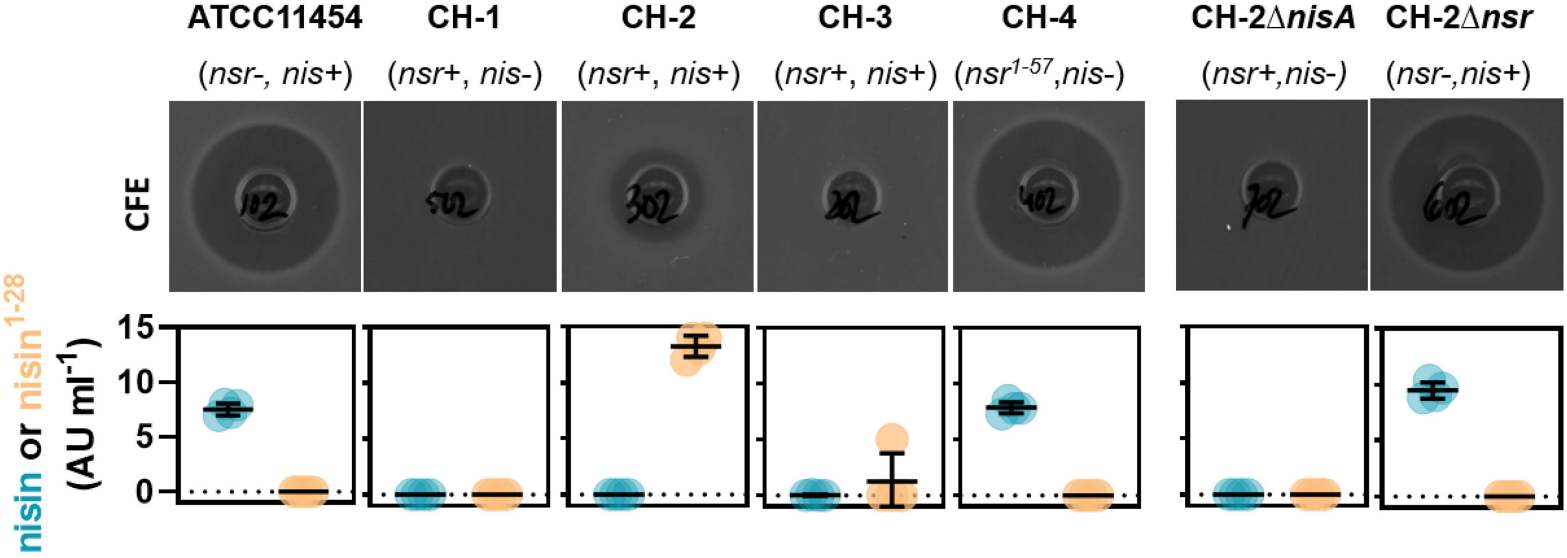
Impact of *nsr* genotype on bactericidal activity and nisin production. Top row: Well diffusion assays showing the antibacterial activity of CFS collected from indicated strains grown in GM17 for 16 h against the nisin-sensitive *L. lactis* strain Wg2. Bottom row: Graphs displaying nisin A and nisin^1−28^ concentrations in CFS collected from indicated strains grown in GM17 for 24 h as measured using HPLC-MS/MS.

We next aimed to study how much nisin A is broken down into nisin^1−28^ in a co-culture containing both a nisin-degrading and a nisin-producing strain. To do so, we employed ATCC 11454 and CH-1 as control as well as three different *nisA*+ *nsr*+ strains. CH-2 (*nisA*+ *nsr*+) has the same genetic make-up as CH-1 but has received the sucrose-nisin transposable element from ATCC 11454 *via* conjugation. CH-3 (*nisA*+ *nsr*+) also possesses an intact *nsr* gene, while CH-4 was found to naturally contain a point mutation in the *nsr* gene, introducing a stop codon at amino acid position 58 yielding a truncated NSR protein. To validate that the possession of *nisA* or *nsr* alone is responsible for nisin synthesis or nisin degradation, a clean deletion of either *nisA* or *nsr* was generated in CH-2, resulting in strains CH-2Δ*nisA* and CH-2Δ*nsr*, respectively. CFS of the seven *L. lactis* strains grown to stationary phase in a rich nutrient broth (GM17) was collected and tested for antibacterial activity by means of well diffusion assays using a nisin-sensitive *L. lactis* strain as indicator. In addition, the presence of nisin A and nisin^1−28^ was assessed using the HPLC-MS/MS method described above. As expected, CFS of strains producing both nisin and NSR displayed a reduced activity against the nisin-sensitive indicator strain (Figure 5). In agreement, large amount of nisin^1−28^ degradation products and no full-length nisin peptides were detected in the CFS of CH-2 and CH-3 (Figure 5). CFS of CH-4 (*nisA*+ *nsr*^1−57^) showed the same level of inhibition as ATCC 11454 (Figure 5). In addition, nisin^1−28^ could not be detected by HPLC-MS/MS, confirming the loss in function of truncated NSR^1−57^. Deleting *nisA* in CH-2 effectively ceased the production of nisin A and thereby also any degraded nisin^1−28^, while deleting *nsr* in CH-2 abolished the presence of nisin^1−28^ in the CFS (Figure 5). However, nisin levels were similar to those detected for ATCC 11454 in CH-2Δ*nsr*, confirming that NSR breaks down nisin produced by *nisA*+ *nsr*+ strains. Together, this data confirms that full-length NSR suffices for nisin degradation.

### Screening of *L. lactis* Strains for Nisin Degradation

**Figure 6:**
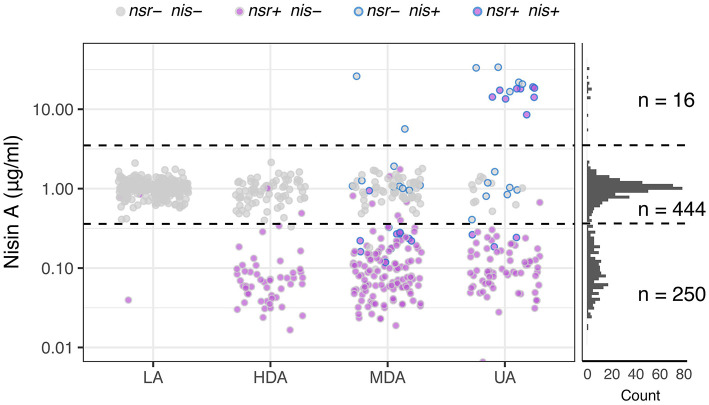
Nisin degradation by individual *L. lactis* strains. Dot plots depicting nisin A concentrations as quantified with HPLC-MS/MS after incubating 0.9 μg ml^−1^ of nisin A in milk inoculated with *L. lactis* strains belonging to one of the four phenotypic groups LA, HDA, MDA and UA. Dots are further color-coded to indicate the presence or absence of *nsr* and/or nisin biosynthesis genes (*nis*) in the tested strain. The right panel displays the distribution of resultant nisin A concentrations derived from the complete set of tested strains (*n* = 710). Horizontal dashed lines are drawn to indicate the boundaries between observations showing an increase of nisin A (16 datapoints above 3.5—upper dashed line), an unchanged nisin A concentration (444 datapoints between dashed lines) or a decreased nisin concentration (250 datapoints below 0.36—lower dashed line).

After establishing that functional NSR is responsible for nisin degradation, we spiked milk with 0.9 μg mL^−1^ nisin A and determined changes in its concentration after fermentation for 18 h with each of the 710 *L. lactis* strains. This allowed us to map the impact of each strain on nisin: Either nisin concentrations increased, remained unaltered or decreased. We used this information to further divide the established phenotypic groups based on acidification profiles into a 3-by-4 phenotypic matrix (Figure 6). In addition, we plotted nisin degradation per variation of the NSR protein sequence to screen for possible inactive variants (Supplementary Figure 6).

The top row of the matrix displayed in Figure 6 shows those strains that led to an increase in nisin A levels above the initial nisin A concentration. As expected, all 16 strains in this group contain the complete nisin A biosynthesis gene cassette and have either an UA or an MDA phenotype (Figure 6). Interestingly, we observe a >5-fold increase in the levels of nisin A of the eight *nsr*+ strains with a nisin biosynthesis cassette, indicating that the nisin production rate by these strains is higher than their nisin degradation rate (Figure 6). It should be noted that, out of 45 strains that contain a full nisin biosynthesis cassette, 29 encode versions other than nisin A that are not detected in the employed HPLC-MS/MS method. As a consequence, these will show up in the middle or bottom row instead.

In the middle row of Figure 6, the largest number of strains can be found. In milk fermentations carried out with these strains, the nisin A concentration remained stable. These largely correspond to strains that do not have the *nsr* gene (430 out of 444). Note that strains mapping to the LA phenotype typically fail to degrade nisin A (Figure 6). Fourteen *nsr*+ strains did not show nisin-degrading activity. Further examination of the NSR protein sequences of these strains revealed 13 different NSR variants (Supplementary Figure 6). Six of these variants were also present in strains with degradation activity, while strains possessing the remaining seven variants showed no indication of nisin degradation. The NSR protein sequences of these seven strains were compared to variant 25, the most abundant form in strains that degrade nisin (also present in CH-1) and a homolog of *S. aureus* NSR of which the structure has been resolved (Khosa et al., 2016) (Supplementary Figure 6). One strain had a N-terminal truncated NSR (variant 21), which likely renders the protease incapable of being transported out of the cell where nisin degradation takes place. For the remaining variants, we found several amino acid substitutions in domains that we speculate abolishes NSR activity (Supplementary Figure 6C) (Khosa et al., 2016).

Strains that map to the bottom row in Figure 6 cause a decline in nisin A, indicative of nisin degradation. Almost all of the 250 milk fermentations (99%) in which a decrease in nisin was observed were carried out by *nsr*+ strains, further strengthening the view that NSR is the fundamental contributor to nisin degradation and hence allows strains to grow and acidify milk in the presence of nisin (Figure 6). We noted in the acidification experiments that strains containing *nsr* as well as nisin immunity showed a bimodal distribution with respect to delay in acidification (Figure 3C). Because NSR variant 17 or 25 were overrepresented in the subgroups with either more or less delay in acidification, respectively, we evaluated their respective nisin degradation capacities when situated in non-nisin producers and found that variant 25 reduced nisin A levels more than variant 17 (Supplementary Figure 6D).

In summary, it is evident from these nisin measurements that nisin degradation by NSR is very common and a mechanism utilized by *L. lactis* strains to protect against nisin.

## Discussion

Nisin-producing strains are added to milk fermentations for cheese production, but how genetic variation in *L. lactis* strains contributes to differences regarding milk acidification remains largely unexplored. We therefore investigated the effect of nisin on milk fermentations from 710 individual *L. lactis* strains. We found that changes in milk acidification by nisin can largely be explained by the presence or absence of nisin immunity (*nisI, nisFEG*) or degradation (*nsr*) genes, in a subspecies dependent manner. Still, within each group a range of strain-specific acidification profiles occurs, which reveal nuances that are easily overlooked in single strain studies focusing on the singular or combinatorial contribution of nisin degradation and/or immunity to nisin tolerance.

### Nisin Degradation Is a Common Trait in Lactococci

Our study identifies nisin degradation by NSR as a very common feature present in 38% of the *L. lactis* strain collection examined (710 strains). In view of these results, nisin degradation *via* NSR appears to be the main mechanism by which lactococci tolerate nisin. Based on this prevalence, we find it likely that one or more *nsr*+ *L. lactis* strains would occur both in natural milk fermentations and undefined starter cultures. In agreement, we have found NSR-specific breakdown products in milk fermented with *L. lactis*-based starter cultures, and in cheese (data not shown). The immunity factors NisFEG and NisI only deliver self-protection, while NSR offers a community-level nisin resistance, cross-protecting also nisin-sensitive strains through the degradation of nisin in the environment. Importantly, the data shows that ssp. *lactis nsr*+ strains are better equipped to degrade nisin in milk than ssp. *cremoris nsr*+ strains.

We believe that NSR activity in a given starter culture is an important determinant for the level by which nisin impacts mixed-strain milk fermentations. On the one hand, NSR presents an advantage by protecting the nisin-sensitive members of the starter culture. On the other hand, a reduction of nisin levels might allow for the growth of undesirable organisms, such as *Clostridia*, which will compete for nutrients and may lead to spoilage of the cheese (Meijer et al., 1998; Sallami et al., 2004). These antitheses pose an important challenge on starter culture design.

### Distribution and Prevalence of Nisin Immunity Genes Vary Between Subspecies

Nisin production and autoimmunity is a trait naturally encountered in strains isolated from raw milk and natural bulk starter cultures (Alegría et al., 2010; Cosentino et al., 2012). Of the *L. lactis* strains examined, 6.3% of the strains contain a full nisin biosynthesis cassette. Thus, this trait is significantly less common than the *nsr* genotype. Nisin synthesis is a property typically assigned to ssp. *lactis* strains, which is further supported by our dataset in which all but one of the nisin-producing strains belong to ssp. *lactis* (De Vuyst, 1994; Alegría et al., 2010; Virolainen et al., 2012). Strikingly, we have identified several nisin producers that also carry the *nsr* gene. At this stage we are unable to put forward an explanation for the role of NSR in nisin-producing strains and we question whether it has a different function that confers a competitive advantage under the right conditions.

Tolerance to nisin can be achieved *via* two mechanisms: degradation with proteases or activity of the immunity proteins NisI and NisFEG. A close inspection of the *L. lactis* collection revealed that about 9% of the strains possessed *nisRK-FEG*, but no synthesis genes. In general, these strains show mild delays in the onset of acidification. The expression of *nisFEG* genes is dependent on nisin-induced activation of NisRK (de Ruyter et al., 1996; Ra et al., 1996). Since *nisRK-FEG* strains do not produce nisin, it is likely that NisFEG proteins are absent in cells of pre-cultures but build up after inoculation in nisin-containing milk. The time that elapses before a sufficient amount of NisFEG is produced for cells to effectively remove nisin from the membrane and resume growth might dictate the severity of the milk acidification lag phase of *nisRK-FEG* strains. Interestingly, *nisRK-FEG* is almost uniquely found in ssp. *lactis* and only present in three ssp. *cremoris* strains. In fact, the unaccompanied *nisIP* operon is the only nisin element specific for ssp. *cremoris* strains. This plasmid-localized operon was shown to confer protection against 20 ng ml^−1^ nisin in ssp. *cremoris* NCDO712 (Tarazanova et al., 2016; Wels et al., 2019). However, the results presented in the current study do not support that NisI alone gives nisin immunity under application-relevant conditions in which nisin concentrations typically range from 1.25 to 7.5 μg ml^−1^ (Davies et al., 1997; Sallami et al., 2004; Aly et al., 2012). Because *nisIP* is only expressed at a low constitutive rate in the absence of nisin-induced *nisABTCIPRK* transcription (Kuipers et al., 1995; de Ruyter et al., 1996; Li and O'Sullivan, 2006; Trmčić et al., 2011), NisI levels are likely to remain lower in *nisIP* strains than in those with the full nisin cassette. NisI quantity directly impacts nisin tolerance due to the fixed 1:1 molar stoichiometry with which it interacts with nisin molecules (Takala et al., 2004; Hacker et al., 2015; Jeong and Ha, 2018). Once nisin outnumbers NisI proteins exposed on the cell surface, the latter can no longer protect nisin from reaching lipid II (AlKhatib et al., 2014a). Taken together, NisI in *nisIP* strains seems to constitute a functional defense mechanism only against low levels of nisin, for instance, when NSR is produced by the same or another strain in the culture. NisFEG in *nisRK-FEG* strains, on the other hand, allows cells to grow in the presence of higher levels of nisin. Like strains that have the full set of immunity genes in combination with NSR, NisFEG or NisI with NSR generally results in an additive effect in nisin tolerance. Given the lack of correlation between the presence of *nisIP* and tolerance to nisin in ssp. *cremoris*, it is reasonable to speculate that NisIP plays a different role in this subspecies.

### Subspecies Are Differently Affected by Nisin During Milk Acidification

Unlike ssp. *lactis*, nisin-tolerant ssp. *cremoris* strains appear to be rare, as substantiated by the following observations derived from our dataset. First, effective nisin immunity and/or resistance seem to be less prevalent in ssp. *cremoris*. Second, ssp. *cremoris* strains that contain *nisFEG* and/or *nsr* generally acidify nisin-containing milk with more delay than ssp. *lactis* of the same genotype. Third, those ssp. *cremoris* strains capable of acidifying milk with a delay have genotypes corresponding to a *lactis* phenotype (Kelleher et al., 2017; Wels et al., 2019). *L. lactis* ssp. *cremoris* strains are used for flavor development of specific cheeses such as cheddar (Broadbent et al., 1998; Børsting et al., 2015). This has been attributed to their ability to lyse, a process that can be accelerated by bacteriocins, thereby releasing intracellular enzymes like peptidases contributing to the flavor during ripening (Morgan et al., 1997). It can thus be argued that the nisin sensitivity of ssp. *cremoris* is desirable and might even have been selected for during its domestication. In general, ssp. *cremoris* are less tolerant than ssp. *lactis* to stress such as elevated temperature, low pH, salt and reactive oxygen species (Kim et al., 1999; Sanders et al., 1999; Dijkstra et al., 2014, 2016). Based on our results, we find that ssp. *cremoris* strains are also less tolerant to nisin.

### Nisin Tolerance Besides Immunity and Degradation

We identified 20 ssp. *lactis* and two ssp. *cremoris* strains that are tolerant (UA or MDA) to nisin, but do not encode any obvious nisin immunity or degradation machineries. We performed a search for homologs of multidrug transporters such as the CprABC-type and BceAB-type systems that are known to confer resistance to nisin in other Gram-positive bacteria, including pathogens and spoilers (Clemens et al., 2018 and references therein), but the results were inconclusive (data not shown). At this stage, we cannot put forward an explanation to why nisin failed to impair acidification of milk by *L. lactis* strains devoid of nisin immunity and degradation pathways. It is well-known that Gram-positive bacteria can adapt to tolerate more nisin (Kramer et al., 2004, 2006, 2008; Giaouris et al., 2008; Bergholz et al., 2013). For instance, adaptive laboratory evolution yielded a ssp. *lactis* strain that could withstand over 75-fold more nisin than its mother strain through temporary transcriptomic alterations (Kramer et al., 2006). Changes in expression were mapped to genes involved in cell wall and phospholipid composition, drug transport and the cell envelope stress response (Kramer et al., 2006; Giaouris et al., 2008). Similar transcriptional responses were also observed in *Staphylococcus aureus, Streptococcus pneumoniae, Listeria monocytogenes*, and *Bacillus subtilis* challenged with nisin (Ming and Daeschel, 1995; Mazzotta and Montville, 1997; Peschel et al., 1999; Hansen et al., 2009; Collins et al., 2010a,b, 2012; Majchrzykiewicz et al., 2010). Moreover, *L. lactis* cells treated with nisin showed overlapping physiological responses to cells challenged with other membrane-perturbing compounds such as Lcn972, another lipid II-binding bacteriocin produced by *L. lactis*, c2 lytic phages, and lysozyme, or by the recombinant production of heterologous membrane proteins (Kramer et al., 2006; Martínez et al., 2007; Veiga et al., 2007; Fallico et al., 2011; Pinto et al., 2011; Roces et al., 2012a,b). In many cases, pre-activating expression of the identified genes improved tolerance toward membrane perturbing compounds including nisin. In order to pinpoint factors involved in the observed subspecies-dependent deviations in innate nisin tolerance, it would be of importance to study nisin-induced changes in gene expression profiles of ssp. *lactis* and ssp. *cremoris* strains that do not have any immunity or degradation machineries in place.

In conclusion, we have characterized a large set of *L. lactis* strains for which we mapped the contribution of nisin immunity and/or degradation genes to nisin tolerance and degradation during milk fermentations. The prevalence of *nsr* and associated nisin degradation is a more common trait than previously reported, albeit the degree of protection established by NSR seems to be highly variable. Full immunity (*nisI* plus *nisFEG*), especially in combination with *nsr*, conferred the best protection. Unlike *nisFEG, nisI* alone hardly delivered immunity under application-relevant conditions. Particularly, nisin impacts ssp. *cremoris* milk acidification more than ssp. *lactis*. Strains that do not produce nisin but have immunity are of great interest for the design of nisin-compatible starter cultures as these would circumvent the requirement for NSR to protect starter culture composition. Such strains have been identified previously and are also found in the current study (Tarazanova et al., 2016; Wels et al., 2019). We believe that the generated dataset lays a new foundation toward understanding how nisin influences cheese fermentation processes that typically involve starter cultures containing multiple nisin-related genotypic variants. Furthermore, the knowledge of how individual strains react to nisin is highly relevant for the design of compatible culture compositions to help fermentation deliver the desired flavor and characteristic properties together with a strong bioprotective effect in the cheese.

## Data Availability Statement

All data is available, except the whole genome sequences, because these are proprietary strains.

## Author Contributions

Experiments were designed by TE, JV, GO, LG, and KN. Experiments were performed by TE, LG, LH-D, AG, and KN. Data was analyzed by LH-D, LG, KN, KJ, EB, TE, AN, and GO. All authors contributed to the first draft with main contributions by LG and TE. Editing and finalization of manuscript was done by LG, LH-D, TE, AN, and GO.

## Conflict of Interest

All authors were employed by CHR HANSEN A/S during the work presented here. CHR HANSEN A/S is a company that develops and commercializes dairy starter cultures.

## Supplementary Material

Supplementary Figure 1 Effect of nisin on milk acidification profiles by 710 *L. lactis* strains co-plotted according to subspecies, phenotypic classification, and genetic make-up of nisin-related gene sets. Milk acidification curves at 30°C of **(A)** *L. lactis* ssp. *lactis* strains and **(B)** *L. lactis* ssp. *cremoris* strains, in the absence (CFS_control_) or presence of 1.5 μg ml^−1^ nisin (CFS_nisin_) and further grouped according to acidification phenotype (rows) and presence or absence of genes for nisin immunity, synthesis, and/or degradation (columns). Thin lines depict averaged milk acidification data of three replicates while thick lines depict the averaged curves per group. The nisin-containing milk phenotypes are: LA, loss of acidification; HDA, highly delayed acidification; MDA, mildly delayed acidification; and UA, unaltered acidification. All acidification curves are synchronized based on acidification onset of each respective CFS_control_ profile and normalized to a start pH of 6.4.

Supplementary Figure 2 Effect of nisin on milk acidification of strains containing a full nisin biosynthesis gene cassette. Graphs depicting milk acidification at 30°C in the absence (CFS_control_) or presence of 1.5 μg ml^−1^ nisin (CFS_nisin_) by strains with *nisABTCIPRK-FEG* genotypes mapping to the **(A)** MDA or **(B)** UA phenotypic group. Each graph depicts the averaged curve of three replicates. Strain IDs as further described in Supplementary Table 1 and subspecies (*lactis* or *cremoris*) are indicated above each pair of curves. All acidification curves are synchronized based on acidification onset of each respective CFS_control_ profile and normalized to a start pH of 6.4.

Supplementary Figure 3 Genotypic diversity of the *L. lactis* strains as revealed by a MLST tree. A MLST-tree depicting the genotypic relatedness of 916 *L. lactis* ssp. *cremoris* and ssp. *lactis* strains. Indicated from outer to inner ring are: **(A)** Strain-specific genome sequence origin, obtained either from the 710 draft genomes from this study or the publicly available 206 *L. lactis* RefSeq genomes. **(B)** Strain-specific presence of nisin-related genes or gene clusters. **(C)** Strain-specific milk acidification phenotypes. **(D)** Subspecies clusters of *L. lactis* ssp. *lactis* and *L. lactis* ssp. *cremoris*.

Supplementary Figure 4 The presence of *nisI* as the sole nisin tolerance factor is not sufficient to deliver protection of ssp. *cremoris* strains to dairy-relevant nisin concentrations. Single replicates of milk acidification profiles of *L. lactis* ssp. *cremoris* strains with *nsr- nisIP*+ *nisFEG-* genotypes in the absence (CFS_control_), presence of 0.2 μg ml^−1^ nisin or presence of 1.5 μg ml^−1^ (CFS_control_). All acidification curves are synchronized based on acidification onset of each respective CFS_control_ profile and normalized to a start pH of 6.4.

Supplementary Figure 5 The presence of *nsr* leads to greater nisin tolerance in ssp. *lactis* strains than in ssp. *cremoris* strains. Upper panels: Milk acidification profiles of ssp. *cremoris* and ssp. *lactis nsr*+ strains in the absence (CFS_control_) or presence (CFS_nisin_) of 1.5 μg ml^−1^ nisin. Thin lines depict the averaged curve between replicates of the same strains while thick lines depict the averaged acidification of each genotypic group. All acidification curves are synchronized based on acidification onset of each respective CFS_control_ profile and normalized to a start pH of 6.4. Lower panels: Subspecific distributions of *nsr*+ strains over acidification phenotypes LA, HDA, MDA, and UA per subspecies.

Supplementary Figure 6 Nisin A levels by NSR variant and subspecies show different protection efficiency. Dot plots depicting resultant nisin A concentrations as quantified with HPLC-MS/MS after incubating 0.9 μg ml^−1^ of nisin A in milk inoculated with **(A)** *L. lactis* ssp. *cremoris* or **(B)** *L. lactis* ssp. *lactis*, as a function of the NSR variant detected in each strain. Dots are further coded according to the phenotypic group (LA, HDA, MDA, and UA) each strain belongs (colors) and if the *nsr* gene is present alone or accompanied by nisin biosynthesis genes (shapes). NSR variants highlighted in green or blue/red indicate most common or non-nisin-degrading NSR variants, respectively. **(C)** Protein sequence alignments of the second-most common NSR variant 17 and non-degrading NSR variants with the most common and functional NSR variant 25 from CH-2. Color-shaded regions of the query sequence of NSR variant 25 indicate domains important for NSR catalytic activity and/or nisin binding reported for the structure function of NSR from *S. agalactiae* (Khosa et al., 2016). **(D)** Box and raincloud plot depicting the difference in resultant nisin A levels in milk fermentations performed with non-nisin A producing strains containing either NSR variant 17 or 25 (Mann-Whitney *U, P* < 0.01).

Supplementary Table 1 List of strains, their genotypes and milk acidification phenotypes, used in this study.

Supplementary Table 2 Query sequences used for gene blasts to identify nisin biosynthesis, immunity and degradation genes.

Supplementary Table 3 Details on data processing steps.

Supplementary Table 4 Primers used in this study.

Supplementary Table 5 Separate milk acidification profiles for all 710 *L. lactis* strains in the presence and absence of nisin.

Supplementary File 1 Sequences of the 12 conserved genes (*dnaK, fusA, groEL, gyrA, gyrB, ileS, lep, pheS, recA, rpoA, rpoB*, and *rpoC*) for each of the 710 *L. lactis* strains, used for the MLST tree presented in Supplementary Figure 3.
