# Supplementary figures and images for: Gene-Trait Matching and Prevalence of Nisin Tolerance Systems in Lactococus lactis
Source: Front Bioeng Biotechnol. 2021 Mar 3;9:622835. doi: 10.3389/fbioe.2021.622835 (PMC7965974; doi:10.3389/fbioe.2021.622835)

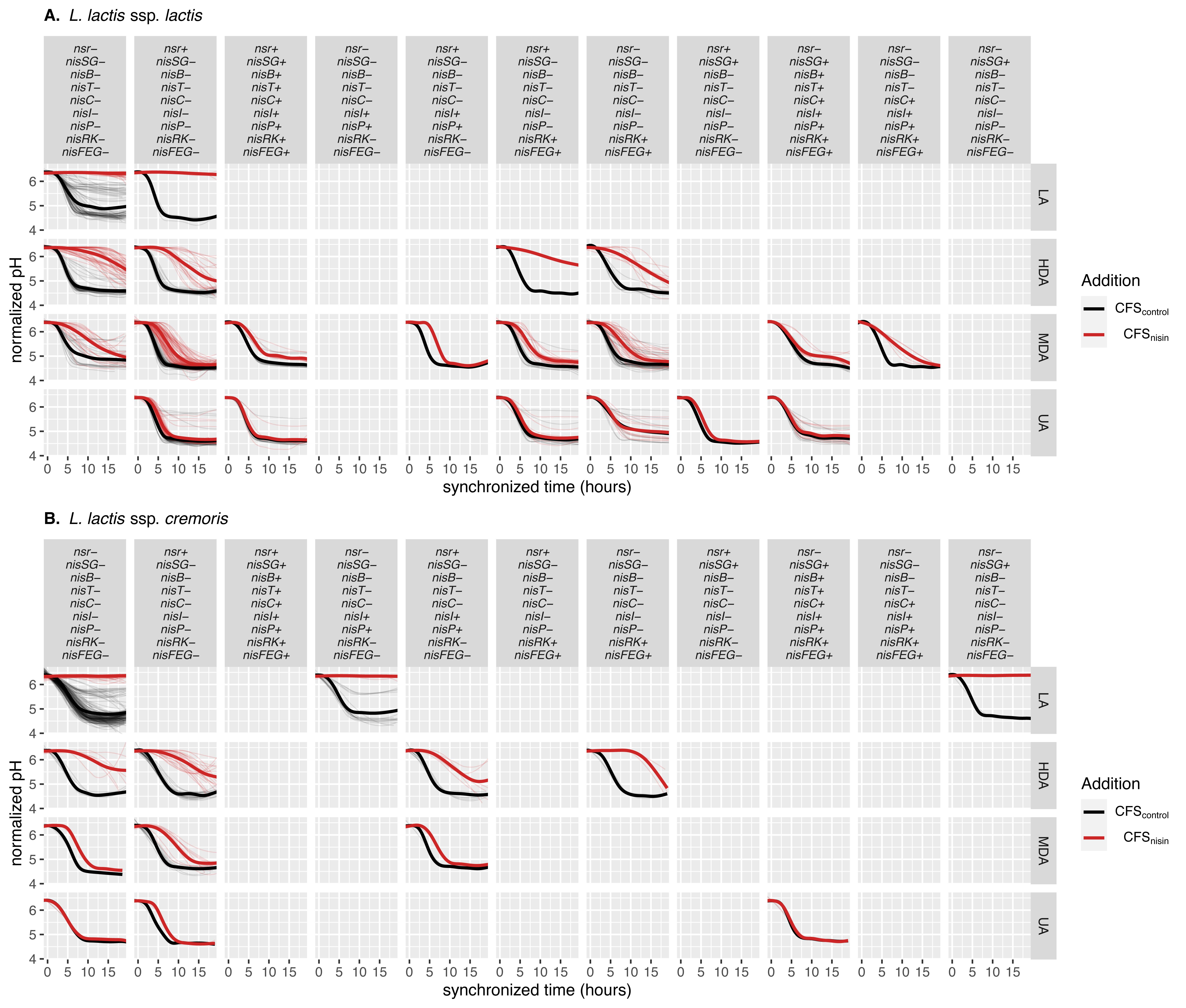

Supplement: Supplementary Figure 1 — Effect of nisin on milk acidification profiles by 710 L. lactis strains co-plotted according to subspecies, phenotypic classification, and genetic make-up of nisin-related gene sets. Milk acidification curves at 30°C of (A) L. lactis ssp. lactis strains and (B) L. lactis ssp. cremoris strains, in the absence (CFScontrol) or presence of 1.5 μg ml−1 nisin (CFSnisin) and further grouped according to acidification phenotype (rows) and presence or absence of genes for nisin immunity, synthesis, and/or degradation (columns). Thin lines depict averaged milk acidification data of three replicates while thick lines depict the averaged curves per group. The nisin-containing milk phenotypes are: LA, loss of acidification; HDA, highly delayed acidification; MDA, mildly delayed acidification; and UA, unaltered acidification. All acidification curves are synchronized based on acidification onset of each respective CFScontrol profile and normalized to a start pH of 6.4. [file Image_1.JPEG]

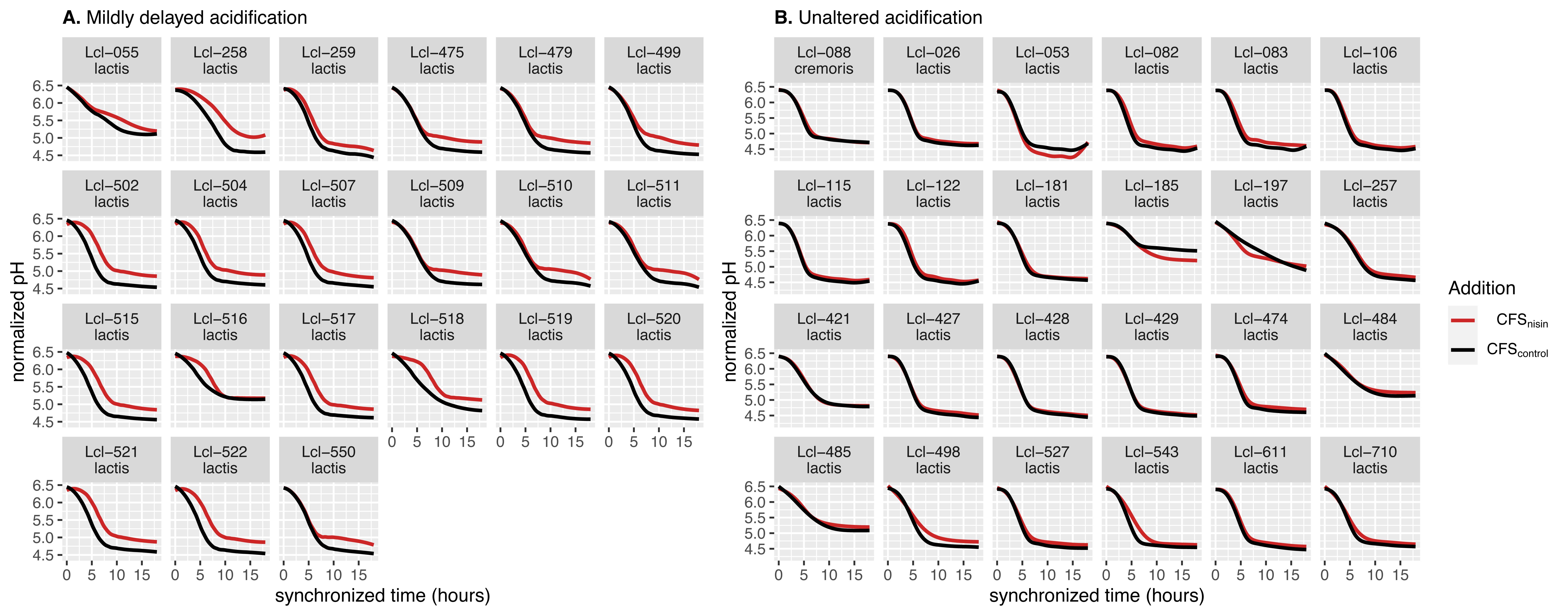

Supplement: Supplementary Figure 2 — Effect of nisin on milk acidification of strains containing a full nisin biosynthesis gene cassette. Graphs depicting milk acidification at 30°C in the absence (CFScontrol) or presence of 1.5 μg ml−1 nisin (CFSnisin) by strains with nisABTCIPRK-FEG genotypes mapping to the (A) MDA or (B) UA phenotypic group. Each graph depicts the averaged curve of three replicates. Strain IDs as further described in Supplementary Table 1 and subspecies (lactis or cremoris) are indicated above each pair of curves. All acidification curves are synchronized based on acidification onset of each respective CFScontrol profile and normalized to a start pH of 6.4. [file Image_2.JPEG]

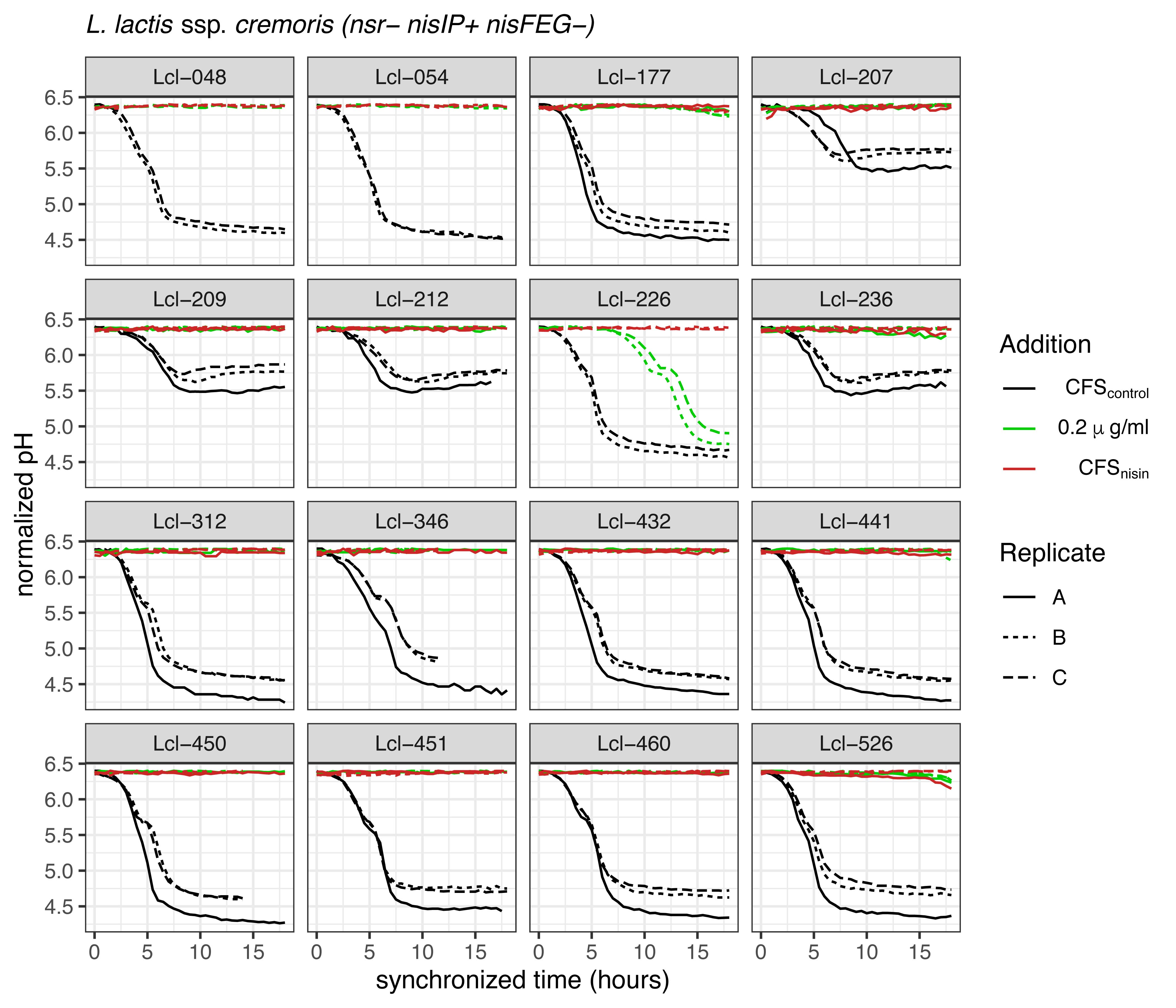

Supplement: Supplementary Figure 4 — The presence of nisI as the sole nisin tolerance factor is not sufficient to deliver protection of ssp. cremoris strains to dairy-relevant nisin concentrations. Single replicates of milk acidification profiles of L. lactis ssp. cremoris strains with nsr- nisIP+ nisFEG- genotypes in the absence (CFScontrol), presence of 0.2 μg ml−1 nisin or presence of 1.5 μg ml−1 (CFScontrol). All acidification curves are synchronized based on acidification onset of each respective CFScontrol profile and normalized to a start pH of 6.4. [file Image_4.JPEG]

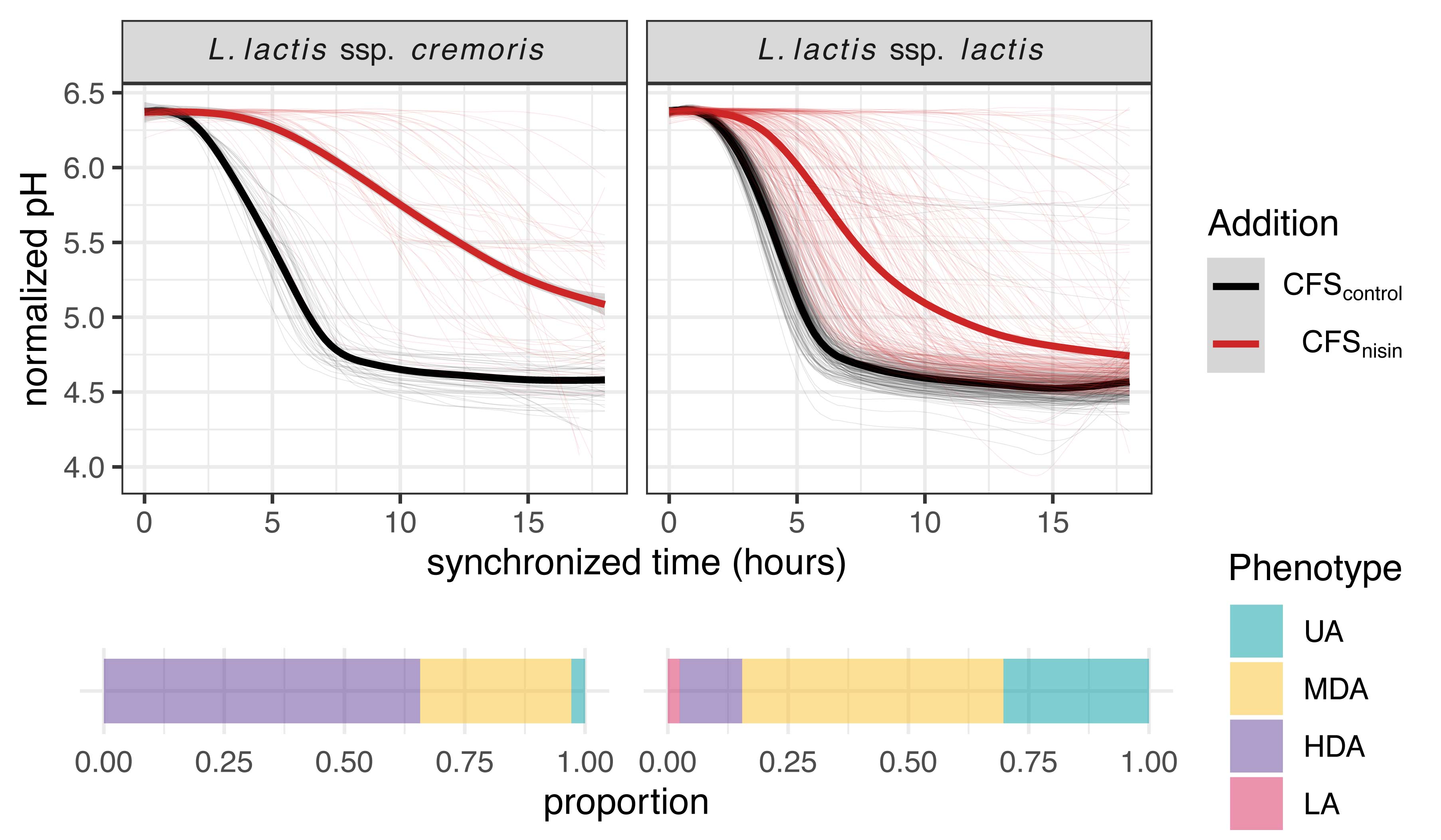

Supplement: Supplementary Figure 5 — The presence of nsr leads to greater nisin tolerance in ssp. lactis strains than in ssp. cremoris strains. Upper panels: Milk acidification profiles of ssp. cremoris and ssp. lactis nsr+ strains in the absence (CFScontrol) or presence (CFSnisin) of 1.5 μg ml−1 nisin. Thin lines depict the averaged curve between replicates of the same strains while thick lines depict the averaged acidification of each genotypic group. All acidification curves are synchronized based on acidification onset of each respective CFScontrol profile and normalized to a start pH of 6.4. Lower panels: Subspecific distributions of nsr+ strains over acidification phenotypes LA, HDA, MDA, and UA per subspecies. [file Image_5.JPEG]

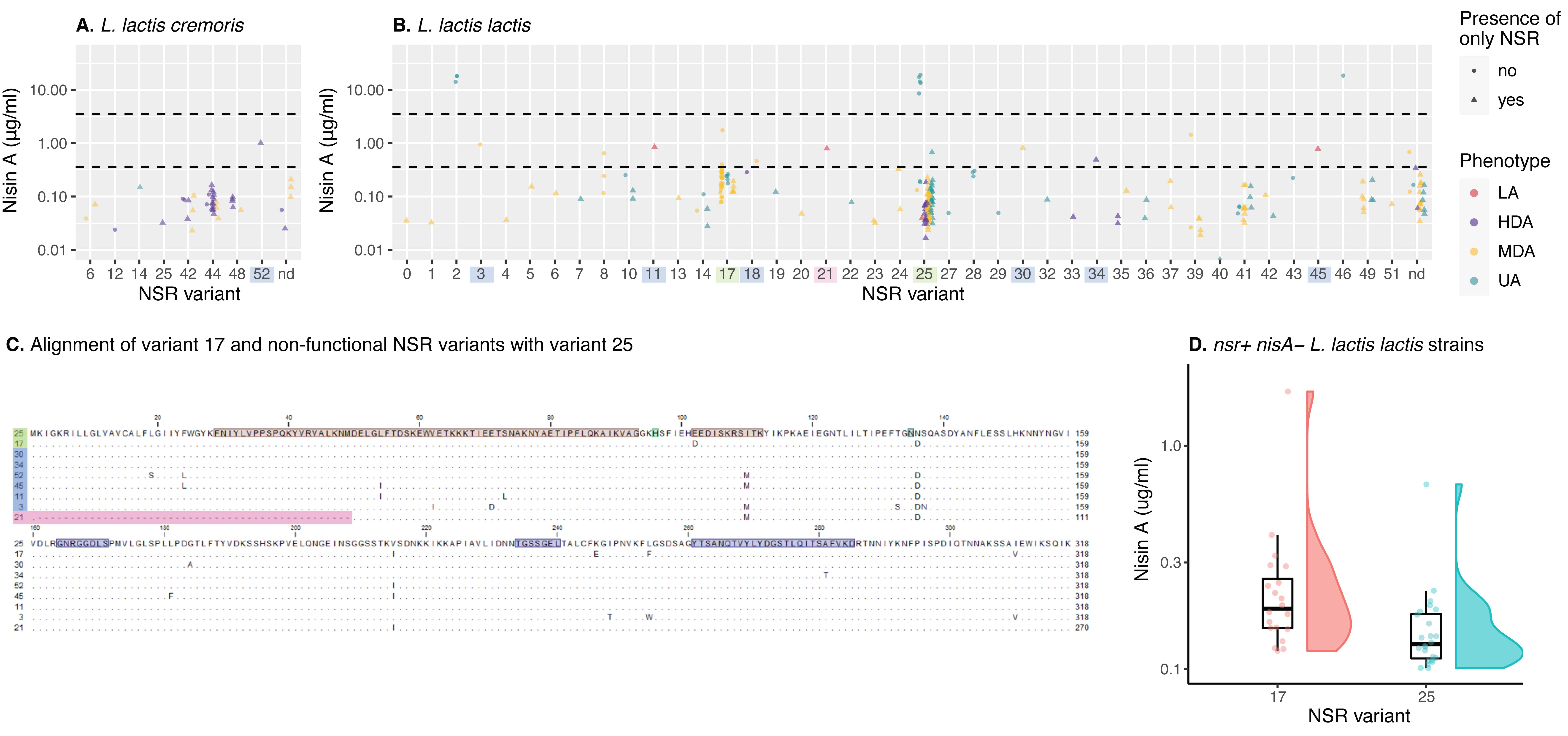

Supplement: Supplementary Figure 6 — Nisin A levels by NSR variant and subspecies show different protection efficiency. Dot plots depicting resultant nisin A concentrations as quantified with HPLC-MS/MS after incubating 0.9 μg ml−1 of nisin A in milk inoculated with (A) L. lactis ssp. cremoris or (B) L. lactis ssp. lactis, as a function of the NSR variant detected in each strain. Dots are further coded according to the phenotypic group (LA, HDA, MDA, and UA) each strain belongs (colors) and if the nsr gene is present alone or accompanied by nisin biosynthesis genes (shapes). NSR variants highlighted in green or blue/red indicate most common or non-nisin-degrading NSR variants, respectively. (C) Protein sequence alignments of the second-most common NSR variant 17 and non-degrading NSR variants with the most common and functional NSR variant 25 from CH-2. Color-shaded regions of the query sequence of NSR variant 25 indicate domains important for NSR catalytic activity and/or nisin binding reported for the structure function of NSR from S. agalactiae (Khosa et al., 2016). (D) Box and raincloud plot depicting the difference in resultant nisin A levels in milk fermentations performed with non-nisin A producing strains containing either NSR variant 17 or 25 (Mann-Whitney U, P < 0.01). [file Image_6.JPEG]
